# Human Amnion Epithelial Cells (AECs) Respond to the FSL-1 Lipopeptide by Engaging the NLRP7 Inflammasome

**DOI:** 10.3389/fimmu.2020.01645

**Published:** 2020-08-07

**Authors:** Marilyne Lavergne, Corinne Belville, Héléna Choltus, Christelle Gross, Régine Minet-Quinard, Denis Gallot, Vincent Sapin, Loïc Blanchon

**Affiliations:** ^1^Genetics, Reproduction and Development (GReD) Laboratory, Clermont Auvergne University, CNRS UMR 6293, INSERM U1103, Translational Approach to Epithelial Injury and Repair Team, Clermont-Ferrand, France; ^2^CHU Clermont-Ferrand, Medical Biochemistry and Molecular Biology Department, Clermont-Ferrand, France; ^3^CHU Clermont-Ferrand, Obstetrics and Gynecology Department, Clermont-Ferrand, France

**Keywords:** fetal membranes, amnion epithelial cells, inflammation, NLRP7, inflammasomes, FSL-1, mycoplasmas

## Abstract

**Context and Objectives:** Inflammation is the leading mechanism involved in both physiological and pathological rupture of fetal membranes. Our aim was to obtain a better characterization of the inflammasome-dependent inflammation processes in these tissues, with a particular focus on the nucleotide-binding oligomerization domain (NOD)–like receptor, pyrin domain containing protein 7 (NLRP7) inflammasome.

**Methods:** The presence of NLRP7 inflammasome actors [NLRP7, apoptosis-associated speck–like protein containing a CARD domain (ASC), and caspase-1] was confirmed by reverse transcriptase–polymerase chain reaction (RT-PCR) in human amnion and choriodecidua at the three trimesters and at term. The protein concentrations were then determined by enzyme-linked immunosorbent assay in term tissues, with or without labor. The presence of *Mycoplasma salivarium* and *Mycoplasma fermentans* in human fetal membranes was investigated using a PCR approach. Human amnion epithelial cells (AECs) were treated for 4 or 20 h with fibroblast-stimulating lipopeptide-1 (FSL-1), a *M. salivarium*–derived ligand. Transcripts and proteins quantity was then measured by RT–quantitative PCR and Western blotting, respectively. NLRP7 and ASC colocalization was confirmed by immunofluorescence. Western blots allowed analysis of pro–caspase-1 and gasdermin D cleavage.

**Results:** NLRP7, ASC, and caspase-1 transcripts were expressed in both sheets of human fetal membranes during all pregnancy stages, but only ASC protein expression was increased with labor. In addition, *M. salivarium* and *M. fermentans* were detected for the first time in human fetal membranes. NLRP7 and caspase-1 transcripts, as well as NLRP7, ASC, and pro–caspase-1 protein levels, were increased in FSL-1–treated AECs. The NLRP7 inflammasome assembled around the nucleus, and pro–caspase-1 and gasdermin D were cleaved into their mature forms after FSL-1 stimulation.

**Conclusion:** Two new mycoplasmas, *M. salivarium* and *M. fermentans*, were identified in human fetal membranes, and a lipopeptide derived from *M. salivarium* was found to induce NLRP7 inflammasome formation in AECs.

## Introduction

Human fetal membranes, which delineate the amniotic cavity during pregnancy, are composed of two sheets: the amnion, which is in contact with the amniotic fluid, and the chorion, which lines the decidua (1). These membranes normally weaken during the last weeks of pregnancy, leading to their physiological rupture at term (2). This weakening occurs in a specific area overlying the cervix, called the “zone of altered morphology” (ZAM), as opposed to the “zone of intact morphology” (ZIM) (3). Many studies have focused on deciphering the phenomena that underlie the weakening of fetal membranes, regardless of whether the ZAM area was distinguished. These phenomena include apoptosis (4–6), extracellular matrix remodeling (7–9), oxidative stress (10, 11), senescence (12–15), epithelial to mesenchymal transition (16), and inflammation (17–20).

Of these phenomena, inflammation is of primary importance for fetal membrane weakening, but it also is required for induction and propagation of prolabor signals. Indeed, the Menon laboratory proposed a model in which reactive oxygen species, increasing in the amniotic cavity at term, induce global senescence of amnion epithelial cells (AECs). These cells acquire a senescence-associated secretory phenotype (SASP) that promotes inflammation by the secretion of specific molecules. Tissue injury, caused by senescence and inflammation, then leads to the release of damage-associated molecular patterns (DAMPs) or alarmins. The SASP and DAMPs contribute to the overall inflammatory load in fetal membranes, and this inflammation propagates to adjacent tissues (chorion, decidua, myometrium, and cervix) to trigger labor (21–24).

This model describes a type of inflammation called “sterile inflammation,” which is an inflammation that is not induced by a pathogen but instead by endogenous molecules released from dead cells. These molecules can include high-mobility group box 1 (HMGB1) protein, cell-free DNA and RNA, interleukin 33 (IL-33), heat shock protein 70, and uric acid (25–27). Sterile inflammation is known to occur in several diseases, such as atherosclerosis (28), gout (29), and ischemia–reperfusion injury (30), as well as in physiological contexts, such as fetal membrane rupture and parturition, as already mentioned.

Sterile inflammation involves inflammasomes as one of the molecular actors. Inflammasomes are cytoplasmic multiprotein platforms composed of (i) a receptor or sensor molecule, (ii) the adaptor protein “apoptosis-associated speck–like protein containing a CARD domain” (ASC), and (iii) the pro–caspase-1 (31–35). The sterile proinflammatory ligands activate and promote the assembly of inflammasomes in a complex called “speck,” which induces the autocatalytic cleavage of pro–caspase-1 into its active form (36). Caspase-1 then cleaves the pro–IL-1β and pro–IL-18 into their bioactive forms (37) and the gasdermin D proteins, whose N-terminal fragments form plasma membrane pores that are associated with pyroptotic cell death (38, 39). The goal of this sterile inflammation process is the propagation of inflammatory signals that will recruit immune cells. The type and origin of the proinflammatory ligands determine which type of sensor molecules is recruited. Our work is focused on one kind of sensor: the nucleotide-binding oligomerization domain (NOD)–like receptor, pyrin domain containing (NLRP) proteins.

The importance of inflammasomes in both normal and pathological pregnancy deliveries has now been demonstrated in several studies. For example, women who underwent spontaneous labor at term showed greater concentrations of ASC and gasdermin D in amniotic fluid when compared to women who delivered without labor (40, 41), and the amount of ASC/caspase-1 protein complexes increased in the fetal membranes (42). Similarly, HMGB1-treated fetal membranes exhibited increased expression of inflammasome-related transcripts (43). These results reflect the inflammasome-dependent preparation of fetal membranes for rupture at term. From a pathological perspective, mouse studies have demonstrated the activation of NLRP3 inflammasomes in fetal membranes after a treatment with the DAMP S100B (44) or lipopolysaccharide (LPS) (45) and a correlated increase in the rate of induced preterm births. This consequence can be decreased by the use of an NLRP3 inhibitor. In women, chorioamnionitis has been associated with an upregulation of inflammasome components, and LPS-treated human amnion cells were able to form ASC specks/filaments (40, 46). A link between NLRP3 inflammasomes and preeclampsia has also been proposed by several groups (47–51).

NLRP7 is a member of NLRP subfamily and is especially known for the involvement of its mutated forms in recurrent hydatidiform moles (52–54). Several studies have also demonstrated a role for NLRP7 in different mechanisms related to pregnancy, including the *in vitro* decidualization of endometrial stromal cells (55), trophoblast lineage differentiation (56), and trophoblast proliferation, migration, and invasion (57). Khare et al. (58) who demonstrated an ASC-dependent caspase-1 activation by NLRP7 in response to microbial acetylated lipopeptides, have first characterized the NLRP7 inflammasome. Zhou et al. (59) also demonstrated an NLRP7 transcription in response to a bacterial infection. These works thus suggest that NLRP7 inflammasomes are activated by pathogens.

The NLRP7 inflammasome has not yet been studied in human fetal membranes. The aim of the present work was therefore to characterize the expression profile of NLRP7 inflammasome actors in human amnion and choriodecidua sheets (i) at the three trimesters, (ii) at term, in samples collected from women who either underwent elective cesarean or delivered vaginally, and (iii) in samples collected from ZAM and ZIM areas. We also investigated the presence of *Mycoplasma salivarium* and *Mycoplasma fermentans*—whose synthetic derivatives are known to activate NLRP7 inflammasomes (58)—in human fetal membranes. Finally, we focused our attention on the mobilization and activation of NLRP7 inflammasome proteins in human AECs in response to fibroblast-stimulating lipopeptide-1 (FSL-1), a synthetic proinflammatory lipopeptide derived from a lipoprotein of *M. salivarium* and known to specifically activate NLRP7 inflammasomes (58).

## Materials and Methods

### Fetal Membrane Collection

The Institutional Local Ethics Committee of the University Hospital of Clermont-Ferrand (specialized for human clinical questions) approved this study and the research protocol. Healthy fetal membranes were collected after receiving oral informed consent (according to the French law named “Huriet-n°88-1138,” which considers placenta and fetal membranes as chirurgical wastes) from the patients in the “Centre Hospitalier Universitaire Estaing” (Clermont-Ferrand, France). The selected fetal membranes were collected from singleton pregnant women who had no underlying diseases or chorioamnionitis.

We collected fetal membranes from the first, second, and third trimesters. The first-trimester fetal membranes (*n* = 3 patients) were obtained and isolated by aspiration after voluntary termination of pregnancy. The second-trimester fetal membranes were harvested after medical termination of pregnancy (*n* = 4 patients). Eligible cases corresponded to lethal fetal anomalies without impact on fetal membranes (e.g., severe cardiac anomalies or brain damage). Third-trimester fetal membranes (*n* = 4 patients) were collected from pregnancies after cesarean births. For second- and third-trimester samples, the amnion was dissociated from the choriodecidua. This was not possible for the first-trimester samples because these two sheets were not yet distinguishable. Term fetal membranes were also collected from women: (i) after spontaneous labor and vaginal deliveries (*n* = 6 patients) and (ii) after scheduled cesarean deliveries prior to labor (*n* = 5 patients). We also collected term fetal membranes (*n* = 5 patients) from women after scheduled cesarean deliveries and prior to labor, where a thread, sewn onto the fetal membranes in front of the cervix, distinguished the ZAM (with the thread) and the ZIM (away from the thread). The amnion was also dissociated from the choriodecidua, thereby leading to the harvest of four kinds of samples: amnion ZAM, amnion ZIM, choriodecidua ZAM, and choriodecidua ZIM.

All the samples were washed four times in 1X Dulbecco phosphate-buffered saline (DPBS; Gibco, 141901169), and the remaining blood vessels were removed manually. Samples of the amnion and choriodecidua (1 cm^2^) were excised and immediately stored at −80°C.

### Enzyme-Linked Immunosorbent Assays

In order to determine protein concentrations of term fetal membranes tissue samples, we used enzyme-linked immunosorbent assay (ELISA) assays. The following kits were used for NLRP7 (MBS9337822; MyBioSource), ASC (MBS7227989; MyBioSource), and caspase-1 (SEB592Hu; Cloud-Clone), according to manufacturer's protocols. Total protein concentration was measured following the protein extraction from fetal membranes explants, and equal quantity of total proteins was added in each ELISA well.

### Human Primary AEC Culture

Delivery products from non-pathological post-cesarean full-term births (37–39 weeks of gestation) were collected at “Centre Hospitalier Universitaire Estaing” (Clermont-Ferrand, France), and the AECs were prepared as previously described (60). Briefly, the amnion was separated from the choriodecidua by peeling and was then washed four times with 1X DPBS. The remaining blood vessels were removed manually. The isolation of human primary AECs was conducted in three trypsinization steps (10, 20, and 30 min; trypsin-EDTA 0.25%; 11560626; Gibco) at 37°C, followed by scraping of the amnion. Cells were filtered to remove the collagen, centrifuged for 5 min at 200 *g*, and grown in T75 flasks coated with collagen type I (04902; Stem Cell Technologies). The AECs were cultivated under standard conditions (5% CO_2_, 95% humidified air, 37°C) in complete Dulbecco modified eagle medium F-12 nutrient mixture (DMEM/F-12, GlutaMAX™ supplement, 31331028; Gibco) supplemented with 10% fetal bovine serum (CVFSVF00-01; Eurobio), 100 μg/mL streptomycin, 100 IU/mL penicillin, and 250 μg/mL amphotericin B (SV30079.01, Hyclone™; GE Healthcare). The absence of mesenchymal cells was checked using vimentin immunolabeling (data not shown). Furthermore, mycoplasma contamination has been checked in culture media (by the Mycoplasmacheck service of Eurofins-Genomics) and was negative. For all experiments, AECs were used after one passage.

### FSL-1 Treatment

Amnion epithelial cells were treated with 250 ng/mL FSL-1 (Pam2CGDPKHPKSF, tlrl-fsl; InvivoGen) and incubated at 37°C for a further 4 or 20 h, depending on the experiment. The cells were then harvested after trypsinization and used for the different experiments. The corresponding data are presented as fold changes relative to the untreated cells, which were set at 1.

### RNA Extraction and Reverse Transcriptase–Quantitative PCR

Total RNA was extracted from fetal membranes explants or primary AECs using the RNeasy® Mini Kit (74106; Qiagen). The RNA concentration was determined by spectrophotometry at 260 nm with a Denovix DS-11 FX spectrophotometer. The cDNA was synthetized from 1 μg RNA using oligo-(dT)_15_ primers (C1101; Promega), 10 mM dNTP (10297-018; Invitrogen), SuperScript IV Reverse Transcriptase (18090050; Invitrogen), and rRNasin® RNase Inhibitor (N2515; Promega), according to the manufacturers' protocols. The primer sequences used for classic or quantitative reverse transcriptase–polymerase chain reaction (qRT-PCR) are detailed in Supplementary Table 1. Recombinant Taq polymerase (10342-020; Invitrogen) and 5 mM dNTP (10297-018; Invitrogen) were used for classic PCR. Negative controls were run without cDNA. Quantitative PCR reactions were performed in duplicate using LightCycler® 480 SYBR Green I Master Mix (04887352001; Roche), according to the MIQE guidelines (61). Standard curves were used to quantify the amount of amplified transcripts. The results were normalized to the geometric mean of the housekeeping genes RPLP0 and RPS17 (“ribosomal protein lateral stalk subunit P0” and “ribosomal protein S17,” respectively). They are given as the ratio between the amount of each transcript of interest and the housekeeping genes' transcripts (consistently expressed over the pregnancy).

### Protein Extraction and Quantification

Fetal membrane tissues or primary AECs were lysed, respectively, with 500 or 250 μL RIPA buffer (20 mM Tris, pH 7.5; 150 mM NaCl; 1% Nonidet P-40; 0.5% sodium deoxycholate; 1 mM EDTA; 0.1% sodium dodecyl sulfate), supplemented with 10% protease inhibitor cocktail (04693159001; Roche). For tissues, a preliminary disruption step was performed using ceramic beads (KT03961-1-009.2; Precellys) and a tissue lyser (Qiagen) (three lysing steps for 25 s with a 30-Hz oscillation frequency, separated by two break steps of 30 s, at 4°C). Samples were then vortex-mixed for 5 s and kept on ice for 10 min; this step was repeated three times. The samples were centrifuged (5 min, 6,800 *g*), and the supernatants were collected. The Pierce™ BCA Protein Assay Kit (23225; Thermo Fisher Scientific) was used to measure the protein concentration according to the manufacturer's guidelines.

### Immunofluorescence Assays and Analysis

Amnion epithelial cells grown on collagen type I–coated coverslips in six-well plates were washed with 1X DPBS and then fixed with 4% paraformaldehyde (15710; Electron Microscopy Sciences) and kept at −80°C. The cells were then permeabilized and blocked with a solution containing 1X DPBS, 4% bovine serum albumin (K41-012; PAA Laboratories), and 0.1% Triton for 1 h at room temperature. The cells were then incubated overnight at 4°C with primary antibodies diluted in blocking solution, as indicated in Supplementary Table 2. After three washing steps with the blocking solution (10 min each), the cells were incubated with a 1:1,000 dilution of donkey Alexa488–anti-rabbit immunoglobulin G (IgG) (A21206; Thermo Fisher Scientific) or donkey Cy3–anti-mouse IgG (715-165-150; Jackson Immunoresearch) antibodies for 2 h at room temperature. The cells were washed again as described earlier, rinsed with 1X DPBS, and incubated with a 1:10,000 dilution of Hoechst (B2883; Sigma-Aldrich) for 20 min at room temperature. Finally, the cells were washed twice with 1X DPBS, and slides were prepared using a mounting medium (Tris-MWL 4-88; Citifluor). The slides were viewed with a LSM 800 microscope (Zeiss), and images were analyzed with ImageJ software (Fiji).

### Western Blotting

The 4–15% gradient TGX polyacrylamide gels (4568084; Bio-Rad) were used for Western blotting. The TGX technology allows absolute quantification of proteins. Thus, protein samples (40 μg) were separated on these gels and transferred to nitrocellulose membranes (1704271; Bio-Rad) using the Trans-Blot® Turbo™ Transfer System (Bio-Rad). After incubation in blocking buffer [5% skim milk in 1X Tris-buffered saline (TBS)] for 2 h at room temperature, the membranes were incubated with primary antibodies diluted in blocking buffer supplemented with 0.05% Tween-20 (Supplementary Table 2) overnight at 4°C. The membranes were washed three times for 10 min with 1X TBS−0.1% Tween-20 (TBST) and incubated with a 1:5,000 dilution of horseradish peroxidase–conjugated anti-mouse IgG or anti-rabbit IgG antibodies (BI 2413C and BI 2407, respectively; Abliance) for 2 h at room temperature. The blots were given three 10-min washes with TBST, rinsed with 1X TBS, and developed on the ChemiDoc™ imaging system (Bio-Rad) with the Clarity Max™ Western ECL Blotting Substrates (1705062; Bio-Rad), according to the manufacturer's protocol. The All Blue Standard (161-0373; Bio-Rad) was used as a protein ladder. Images were analyzed with Image Lab™ software (Bio-Rad). The total protein normalization (Bio-Rad), a method allowing a normalization using the total protein loaded, has been used to normalize the Western blot results.

### *Mycoplasma salivarium* and *Mycoplasma fermentans* Detection

Genomic DNA (gDNA) was extracted with a QIAmp DNA Mini Kit (51306; Qiagen) from amnion and choriodecidua samples collected from women who delivered vaginally (*n* = 7 patients) or by cesarean section (*n* = 7 patients). Specific primers for *M. salivarium* (62) and *M. fermentans* (63) were used to amplify DNA and detect these species (Supplementary Table 3). Polymerase chain reaction was conducted using Q5® High-Fidelity DNA Polymerase (M0491S; New England Biolabs) and 10 or 20 ng of gDNA for *M. salivarium* and *M. fermentans*, respectively. The following amplification program was used: 30 s at 98°C, followed by 40 amplification cycles comprising 10 s at 98°C, 30 s at 62°C or 65°C according to primers pair (Supplementary Table 3), and 30 s at 72°C, ending with 2 min at 72°C. Positive controls were run using commercial gDNA from both mycoplasma species (52-0117 and 52-0103, Minerva Biolabs), according to the manufacturer's instructions. Amplified DNA was visualized on 2% agarose gels prepared with 1X Tris borate electrophoresis buffer and stained with ethidium bromide.

### Statistical Analysis

All data were analyzed using the GraphPad Prism Program version 5.02. The non-parametric Mann–Whitney *U*-test was used to compare two independent groups. When more than two groups were compared, the non-parametric one-way analysis of variance (Kruskal–Wallis) test was applied, followed by multiple comparison with Dunn correction. In all cases, *p* < 0.05 was considered statistically significant.

## Results

### ASC Protein Expression Is Increased in Human Fetal Membranes With Labor

**Figure 1:**
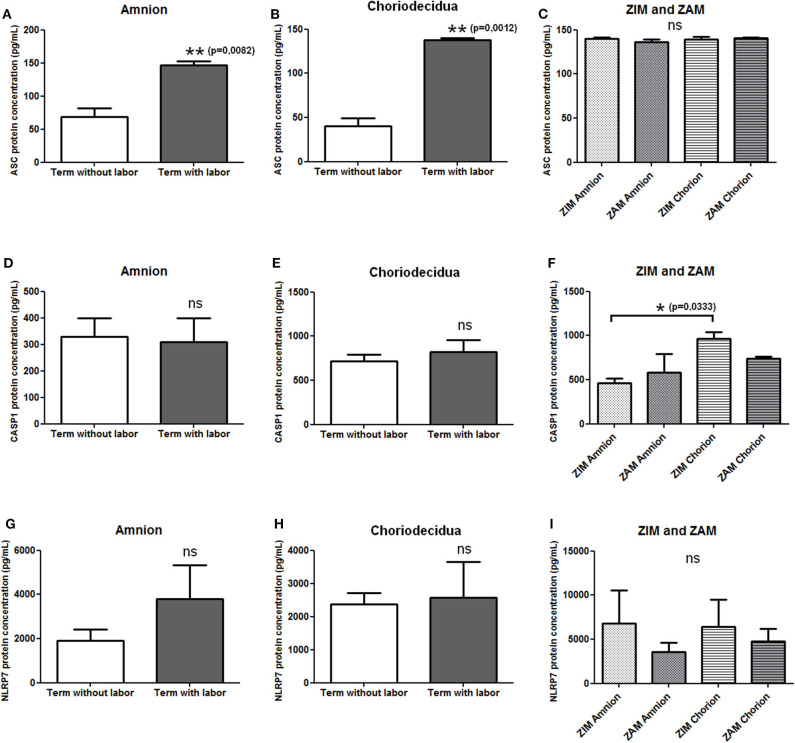
Protein expression level of NLRP7 inflammasome actors in term fetal membranes. Protein expression of ASC **(A–C)**, caspase-1 **(D–F)**, and NLRP7 **(G–I)** was measured in term amnions and choriodeciduas with or without labor **(A,B,D,E,G,H)** and in ZIM and ZAM areas at term **(C,F,I)** using ELISAs. Number of samples: *n* = 7 for term without labor tissues; *n* = 6 for term with labor tissues; *n* = 5 for ZIM and ZAM tissues. Statistics for **(C,F,I)**: non-parametric one-way analysis of variance, Kruskal–Wallis test, Dunn post-test. Statistics for **(A,B,D,E,G,H)**: non-parametric *t*-test, Mann–Whitney *U*-test. ns, not significant. *p < 0.05; **p < 0.01. ASC, apoptosis-associated speck–like protein containing a CARD domain; CASP1, caspase-1; NLRP, nucleotide-binding oligomerization domain-like receptor, pyrin domain containing; ZAM, zone of altered morphology; ZIM, zone of intact morphology.

We first checked the expression of mRNAs of NLRP7 inflammasome actors (i.e., NLRP7, ASC, and caspase-1) in human fetal membranes using RT-PCR, and we observed the expression of these transcripts in amnion and choriodecidua during all pregnancy stages (data not shown). We then determined whether the protein expression of NLRP7, ASC, and caspase-1 in the amnion and choriodecidua was influenced by labor and whether the expression varied between the ZIM and ZAM areas. Enzyme-linked immunosorbent assay were used to quantify protein expression of ASC (Figures 1A–C), caspase-1 (Figures 1D–F), and NLRP7 (Figures 1G–I). Labor affected only the expression of ASC, which was increased in both the amnion and choriodecidua (Figures 1A,B). Caspase-1 (Figures 1D,E) and NLRP7 (Figures 1G,H) protein expression was unchanged with labor. We did not detect any difference in expression between the ZIM and ZAM areas (Figures 1C,F,I), but caspase-1 expression was significantly higher (*p* = 0.033) in the ZIM area of the choriodecidua than in the ZIM area of the amnion (Figure 1F).

### Pregnant Women Are Healthy Carriers of *M. fermentans* and *M. salivarium*

**Figure 2:**
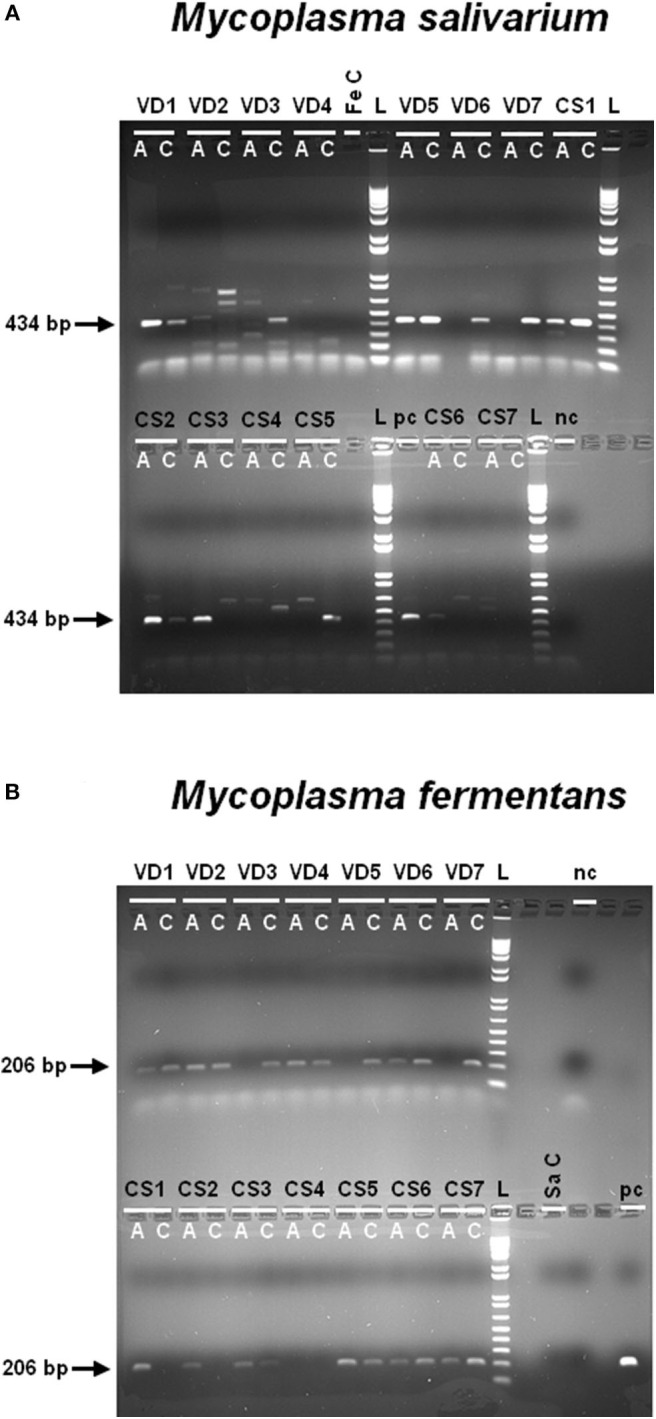
*Mycoplasma salivarium* **(A)** and *Mycoplasma fermentans* **(B)** are present in human fetal membranes. Using specific primers for *M. salivarium* **(A)** or *M. fermentans* **(B)** gDNA (Supplementary Table 3), these mycoplasmas were identified in human amnions (A) and choriodeciduas (C), obtained after vaginal delivery (VD; *n* = 7) or cesarean section (CS; *n* = 7). We checked the primers were not interspecific by amplifying the gDNA of one species with the primers of the other species (Fe C and Sa C, *M. fermentans* gDNA control and *M. salivarium* gDNA control). The PCR products amplified with specific primers for *M. salivarium* or *M. fermentans* have a length of 434 or 206 bp, respectively. The negative control (PCR mix without DNA) and positive control (using *M. salivarium* and *M. fermentans* gDNA) were indicated as nc and pc, respectively. A 2% agarose gel prepared with 1X Tris borate electrophoresis and stained with ethidium bromide was used to visualize the results of PCR. The ladder (L) is 1 Kb Plus DNA Ladder (10787-026; Invitrogen). VD, vaginal delivery; CS, cesarean section; A, amnion; C, choriodecidua; Fe C, specificity control with *M. fermentans* gDNA; Sa C, specificity control with *M. salivarium* gDNA; nc, negative control; pc, positive control; bp, base pair; L, ladder.

We evaluated the presence of *M. fermentans* and *M. salivarium*—known to activate NLRP7 inflammasomes (58)—in term fetal membranes samples obtained with or without labor, by PCR using specific primers (Supplementary Table 3). These mycoplasmas were found in almost all amnion and choriodecidua samples, regardless of whether the women delivered vaginally or by cesarean section (Figures 2A,B). This is the first report of *M. salivarium* in term human fetal membranes. *Mycoplasma fermentans* was previously detected in amniotic fluid (64), but this report is the first to identify this mycoplasma in term human fetal membranes. The samples for this experiment were collected from women without a pathological pregnancy, so we can assume that pregnant women are healthy carriers of *M. salivarium* and *M. fermentans*. Both of which could potentially be involved in the induction of an NLRP7-dependent inflammatory response in fetal membranes and contribute to membrane weakening.

### NLRP7, ASC, and Caspase-1 Proteins Are Increased in AECs in Response to FSL-1

**Figure 3:**
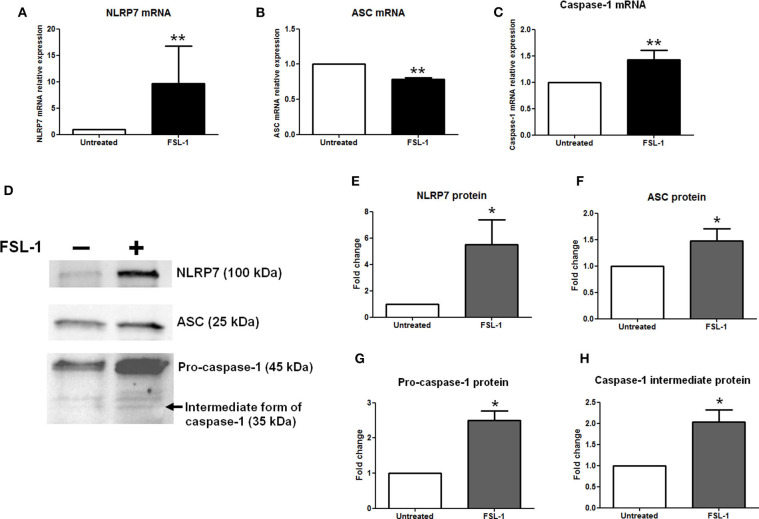
NLRP7, ASC, and caspase-1 expression is increased, as well as the pro–caspase-1 is processed, in response to FSL-1 in AECs. Expression of NLRP7 **(A,D,E)**, ASC **(B,D,F)**, and caspase-1 **(C,D,G,H)** was measured at transcript **(A–C)** and protein **(D–H)** levels with or without FSL-1 treatment, using RT-qPCR (*n* = 6) and Western blotting (*n* = 4), respectively. Representative Western blot data are shown in **(D)**. Transcript and protein levels were evaluated 4 or 20 h after 250 ng/mL of FSL-1 exposure, respectively. Statistics: non-parametric *t*-test, Mann–Whitney *U*-test. ns, not significant. *p < 0.05; **p < 0.01. AECs, amnion epithelial cells; ASC, apoptosis-associated speck–like protein containing a CARD domain; FSL-1, fibroblast-stimulating lipopeptide-1; NLRP, nucleotide-binding oligomerization domain-like receptor, pyrin domain containing.

Primary AECs were harvested from amnions collected after cesarean section and cultured *in vitro*. The expression of NLRP7, ASC, and caspase-1 was measured by RT-qPCR (Figures 3A–C) and Western blotting (Figures 3D–H) after a 4- or 20-h exposure, respectively, to FSL-1 at 250 ng/mL, which is a well-established concentration (65–67). The NLRP7 mRNA and protein levels were significantly increased by FSL-1 treatment when compared with the control condition (Figures 3A,D,E). By contrast, the ASC transcript expression was decreased by FSL-1 treatment (Figure 3B), while its protein level was increased (Figures 3D,F). The processing of pro–caspase-1 protein into its intermediate form (considered as the active form) was also analyzed. Transcripts of caspase-1 (Figure 3C) and the precursor and intermediate proteins of caspase-1 (Figures 3D,G,H) were higher in AECs after FSL-1 treatment than in the untreated condition. Therefore, AECs responded to FSL-1 by increasing the protein expression of NLRP7, ASC, and caspase-1, the three molecular actors involved in the formation of NLRP7 inflammasomes. Furthermore, caspase-1 protein was activated in AECs in response to FSL-1.

### NLRP7 and ASC Proteins Colocalize in AECs After FSL-1 Treatment

**Figure 4:**
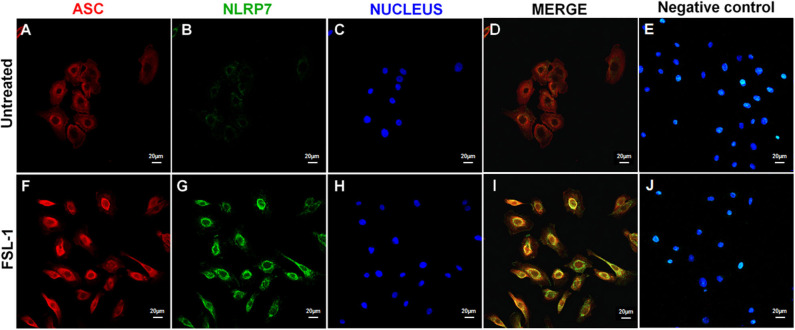
NLRP7 and ASC colocalize in response to FSL-1 in AECs. Immunofluorescence assay of AECs treated **(F–J)** or not **(A–E)** with 250 ng/mL of FSL-1 for 20 h. ASC proteins were stained in red **(A,F)**, NLRP7 proteins in green **(B,G)**, and nucleus in blue with the Hoechst staining **(C,H)**. Merged pictures are seen in D and I without the Hoechst channel in order to better visualize the colocalization of NLRP7 and ASC. Negative controls, corresponding to secondary antibodies incubation without primary antibodies, attested the specificity of antibodies used **(E,J)**. Magnification × 400; with the LSM 800 microscope (Zeiss); *n* = 3. AECs, amnion epithelial cells; ASC, apoptosis-associated speck–like protein containing a CARD domain; FSL-1, fibroblast-stimulating lipopeptide-1; NLRP, nucleotide-binding oligomerization domain-like receptor, pyrin domain containing.

Immunofluorescence assays were conducted to establish the localization of NLRP7 and ASC in AECs 20 h after the FSL-1 treatment (Figure 4). In untreated AECs, NLRP7 staining was almost non-existent (Figure 4B), and the observed ASC staining indicated that this protein was diffusely distributed in the cytoplasm (Figure 4A). Treatment with FSL-1 increased the intensity of the NLRP7 and ASC staining (Figures 4F,G), in agreement with the Western blot results (Figures 3D–F). The ASC proteins were localized around the nucleus and colocalized with the NLRP7 proteins (Figure 4I).

### Gasdermin D Protein Is Overexpressed and Processed After FSL-1 Treatment

**Figure 5:**
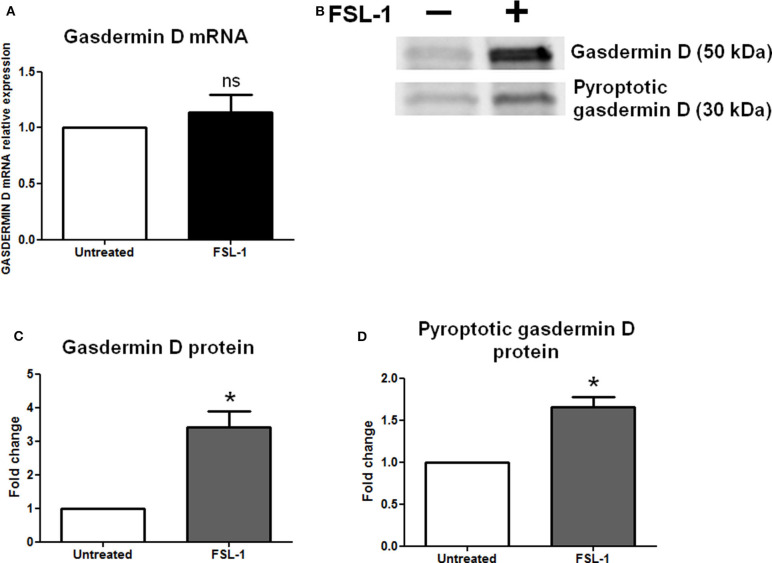
Gasdermin D protein is increased and processed in response to FSL-1 in AECs. Expression of gasdermin D and its pyroptotic form was measured at transcript **(A)** and protein **(B–D)** levels with or without FSL-1 treatment, using RT-qPCR (*n* = 6) and Western blotting (*n* = 4), respectively. Representative Western blot data are shown in **(B)**. Transcript and protein levels were evaluated 4 or 20 h after 250 ng/mL of FSL-1 exposure, respectively. Statistics: non-parametric *t*-test, Mann–Whitney *U*-test. ns, not significant. *p < 0.05. AECs, amnion epithelial cells; FSL-1, fibroblast-stimulating lipopeptide-1.

Inflammasomes induce a classically pyroptotic cell death mediated by the gasdermin D proteins that form pores on plasma membranes to allow the release of cellular contents. We used RT-qPCR and Western blotting to examine the amounts of gasdermin D mRNAs and proteins, respectively, in AECs (Figure 5). The gasdermin D transcript expression was the same in the untreated and FSL-1–treated conditions (Figure 5A); however, the expression of the precursor of gasdermin D protein was increased 20 h after FSL-1 treatment (Figures 5B,C). The pyroptotic form of gasdermin D protein was also increased (Figures 5B,D).

## Discussion

Inflammation is involved in both the normal physiological and the pathological rupture of human fetal membranes during parturition. Recently, particular attention has been paid to inflammasomes and their molecular actors, as these have been linked to preterm prelabor rupture of membranes (pPROM) (68, 69), chorioamnionitis (42, 46, 70), preterm labor (71–73), and preeclampsia (48, 50, 74). Thus, a better characterization of inflammasome-related inflammation seems to be essential in order to prevent potentially adverse pregnancy outcomes.

This work demonstrates for the first time that the transcripts of the three NLRP7 inflammasome actors (NLRP7, ASC, and caspase-1) are expressed in human fetal membranes during all trimesters and at term, meaning that this inflammasome can assemble at all stages of pregnancy in response to pathogen-associated molecular pattern and DAMP production throughout pregnancy. We focused on term physiological rupture of fetal membranes, so we examined the protein expression of these three actors at term, with or without labor, and in the ZAM and ZIM areas.

ASC expression was higher in the amnion with labor at term than without labor, and this has not been reported before. This result is consistent with the observation of Gomez-Lopez et al. (42) of an increased number of ASC/caspase-1 complexes with labor in fetal membranes. We did not observe a change in the expression for caspase-1 protein with labor, in accordance with the work of Romero et al. (75), who also used ELISAs for caspase-1 concentration measurements. In that same study, Western blots revealed no difference in pro–caspase-1 accumulation in fetal membranes with or without labor; however, an increased accumulation of the p20 isoform was observed with labor, as was an increase in caspase-1 immunostaining. Similarly, Lappas (76) demonstrated an unchanged accumulation of pro–caspase-1 protein in fetal membranes by Western blotting, whereas the p35 and p10 isoforms were increased with labor. The NLRP7 protein expression was not affected by labor in the present study. Romero et al. (75) demonstrated that NLRP3 protein expression was increased in fetal membranes with labor, suggesting a specific behavior of each NLRP family member during labor. Thus, among the three NLRP7 inflammasome actors, only ASC protein level is increased in human fetal membranes with labor, which could contribute to the recruitment and activation of inflammasomes at term during parturition. It is worth noting that Romero et al. (75) also measured the protein concentration of IL-1β and IL-18 in human fetal membranes and observed an increased protein expression of IL-1β with labor, compared to unlabored women, whereas the IL-18 protein concentration is unchanged in the same conditions.

To better decipher the NLRP7 protein expression in fetal membranes, we investigated the presence of two known activators of NLRP7 inflammasomes, *M. salivarium* and *M. fermentans*, in the tissues. This study provides the first evidence for *M. salivarium* and *M. fermentans* in the fetal membranes of healthy women. *Mycoplasma fermentans* has previously been found in the amniotic fluid of women who presented chorioamnionitis and villitis (64) and in women's genital tracts (77). The detection of these bacteria in fetal membranes obtained after vaginal delivery could be due to a contamination after fetal crossing through the genital tract. However, these bacteria were also present in samples obtained after cesarean sections, suggesting that *M. salivarium* and *M. fermentans* reach the fetal membranes by genital tract ascension and that some women are healthy carriers. Some mycoplasmas are known to be involved in pathological pregnancies (e.g., chorioamnionitis, preterm labor, pPROM) due to their overgrowth (78–84). As *M. salivarium* and *M. fermentans* were found in term fetal membranes, they could activate NLRP7 inflammasomes in fetal membranes, which could contribute to their weakening at term or at least to the overall inflammatory load observed in this area at term. Therefore, obtaining a better understanding of the molecular pathway induced by mycoplasmas is important.

Special attention should also be paid to the relationship between mycoplasmas and inflammation induced by inflammasomes. We addressed this by examining the mobilization of inflammasomes in response to mycoplasmas, using the known activation of NLRP7 inflammasomes in macrophages by the *M. salivarium*–derived ligand FSL-1 (58). We found that FSL-1 treatment increased the expression of NLRP7 and caspase-1 transcripts and proteins in AECs. Thus, the expression of two of the molecular actors necessary to form NLRP7 inflammasomes was stimulated by FSL-1. This finding reflects the transcriptional priming observed in the canonical pathway of NLRP3 inflammasome activation (85) and suggests that the NLRP7 inflammasome also undergoes a transcriptional priming step. Interestingly, our results could be considered as complementary with two previous works: one of Saeki et al. (86), demonstrating the activation of the NLRP3 inflammasome in murine macrophages treated with FSL-1, and the other of Khare et al. (58) demonstrating the activation of NLRP7 in human macrophages treated with FSL-1. This paves the way to further investigations.

The transcript expression of ASC was decreased, whereas the ASC protein level was increased by FSL-1 treatment. Only one previous study has reported a decrease in the ASC protein level in human monocytes infected with *Legionella pneumophila*; this decreased expression was abolished once ASC expression was dependent on a *L. pneumophila* impervious promoter (87).

Proinflammatory signals induce the formation of inflammasomes, which then gather in a cytosolic macromolecular complex called the “ASC speck” (88). As other NLRP proteins, NLRP7 is able to form an inflammasome. Indeed, this protein is composed of three main domains: the pyrin domain (PYD) at the N-terminus, the leucine-rich repeat (LRR) domain at the C-terminus, and the central nucleotide-binding domain (NBD), containing the NACHT domain (which comes from “present in NAIP, CIITA, HET-E, and TP-1”) adjacent to the NACHT-associated domain (NAD) (89, 90). The NBD binds ATP and exhibits ATPase activity, which is necessary for the NLRP7 oligomerization and inflammasome activation (91). Singer et al. (92) then refined these results and proved that the NLRP7 proteins physically oligomerize through the NAD. Moreover, Khare et al. (58) demonstrated the interaction between the PYD of NLRP7 and ASC, as well as the coimmunoprecipitation of NLRP7, ASC, and caspase-1 in human macrophages infected with *Staphylococcus aureus*. Finally, it is now well-described that the ASC protein binds the NLRP protein and the caspase-1 through homotypic interactions with its PYD and caspase recruitment domain (CARD), respectively (93). In the present study, ASC and NLRP7 were present and colocalized in the AECs in a location around the nucleus. This location of ASC agrees with the results recently obtained by Panaitescu et al. (40), who showed ASC localization all around the nucleus in amnion epithelial and mesenchymal cells in response to LPS and nigericin. Moreover, the colocalization of NLRP7 and ASC around the nucleus suggests an interaction between these proteins and organelles. Indeed, NLRP7 is able to colocalize with the Golgi apparatus and the microtubule-organizing center within human peripheral blood mononuclear cells (89). Other work has demonstrated that NLRP3 and ASC interact with mitochondria and the endoplasmic reticulum in THP-1 cells following stimulation (94). Thus, we suggest that, like the NLRP3 inflammasomes, NLRP7 inflammasomes may also interact with organelles to contribute to their activation and functions in AECs.

Caspase-1 is the executor molecule of inflammasomes (85), and caspase-1 transcripts and pro–caspase-1 protein expression were increased in AECs in response to FSL-1 treatment. We confirmed inflammasome activation by assessing pro–caspase-1 processing by detecting an increased level of caspase-1 intermediate form (p35). This is in agreement with the work of Boucher et al. (36), in which they recently demonstrated in mouse macrophages that the form of caspase-1 with major cleavage activity is the intermediate form (p33/p10 or p35) and not the dimer p20/p10, as was initially supposed for many years.

An *in vivo* study showed that IL-1β mRNA expression is increased in mouse fetal membranes following LPS stimulation (45). Interleukin 1β transcript expression is also increased in term human fetal membranes explants treated with LPS (95). Interleukin 1β transcription is also increased in fetal membranes with labor (75). Thus, an increased expression of IL-1β mRNA in fetal membranes is correlated with both pathological and physiological conditions. In our study, we also demonstrated a similar response of AECs to FSL-1, illustrated by an increased expression of IL-1β transcripts (Supplementary Figure 1) corresponding to the already described “priming step.” Nevertheless, by using three different methods of protein identification and quantification (Western blot, ELISA, and Multiplex assays), we could not detect an increase of the mature IL-1β protein (in cell lysates and culture media). Thus, as other inflammasomes, the NLRP7 inflammasome may request a “second signal” in order to proceed such IL maturation. Activation of inflammasomes also induces gasdermin D cleavage, leading to the formation of membrane pores by the N-terminal fragment, also called the pyroptotic fragment (96). Recently, Gomez-Lopez et al. (41, 73) associated gasdermin D expression and subsequent pyroptosis with both term and preterm deliveries. In the present study, the precursor and pyroptotic forms of gasdermin D were more strongly expressed in AECs following FSL-1 treatment than in the untreated condition. Taken together, our results suggest the formation of gasdermin D pores on AEC plasma membranes—and the subsequent pyroptosis—in absence of IL-1β release, in response to FSL-1.

To conclude, these findings demonstrated that NLRP7 inflammasomes are formed in AEC following stimulation with a lipopeptide derived from *M. salivarium*, suggesting an involvement of NLRP7 in amnion during the physiological rupture of membranes. The amnion and chorion are both involved in fetal membranes rupture; therefore, complementary investigations should be initiated to determine if NLRP7 inflammasomes are also established in chorion cells during this obstetrical phenomenon.

## Data Availability Statement

All datasets generated for this study are included in the article/Supplementary Material.

## Author Contributions

ML designed and performed the experiments and wrote the manuscript. CB, HC, and CG helped ML to carry out some experiments. CB validated the statistical analysis. DG allowed ML to obtain human fetal membranes from patients in the Estaing Hospital (Clermont-Ferrand, France). VS and LB supervised the project. VS, LB, CB, and RM-Q revised the manuscript and contributed to its writing. All authors contributed to the article and approved the submitted version.

## Conflict of Interest

The authors declare that the research was conducted in the absence of any commercial or financial relationships that could be construed as a potential conflict of interest.
