# Supplementary material for: Human Amnion Epithelial Cells (AECs) Respond to the FSL-1 Lipopeptide by Engaging the NLRP7 Inflammasome
Source: Front Immunol. 2020 Aug 7;11:1645. doi: 10.3389/fimmu.2020.01645 (PMC7426397; doi:10.3389/fimmu.2020.01645)
Supplement: Supplementary file 1 [file Data_Sheet_1.pdf]

SUPPLEMENTARY DATA

Human amnion epithelial cells (AECs) respond to the FSL-1 lipopeptide by engaging the NLRP7 inflammasome

Marilyne LAVERGNE[1]*, Corinne BELVILLE[1], Héléna CHOLTUS[1], Christelle GROSS[1], Régine MINET-QUINARD[1,3], Denis GALLOT[1,2], Vincent SAPIN[1,3], Loïc BLANCHON[1†].

[1] Genetics, Reproduction and Development (GReD) Laboratory, Clermont Auvergne University, CNRS UMR 6293, INSERM U1103, Translational Approach to Epithelial Injury and Repair Team, Clermont-Ferrand, France.

[2] CHU Clermont-Ferrand, Obstetrics and Gynecology Department, Clermont-Ferrand, France.

[3] CHU Clermont-Ferrand, Medical Biochemistry and Molecular Biology Department, Clermont-Ferrand, France.

***ORCID:** https://orcid.org/0000-0002-4101-8675

[†]**Correspondence:** Loïc BLANCHON, Centre de Recherche Bioclinique, 28 place Henri DUNANT, TSA 50400, 63001 Clermont-Ferrand Cedex 1 (France); Tel : +33 4 73 17 81 74**;** loic.blanchon@uca.fr. **ORCID:** https://orcid.org/ 0000-0001-8842-0162

SUPPLEMENTARY FIGURE

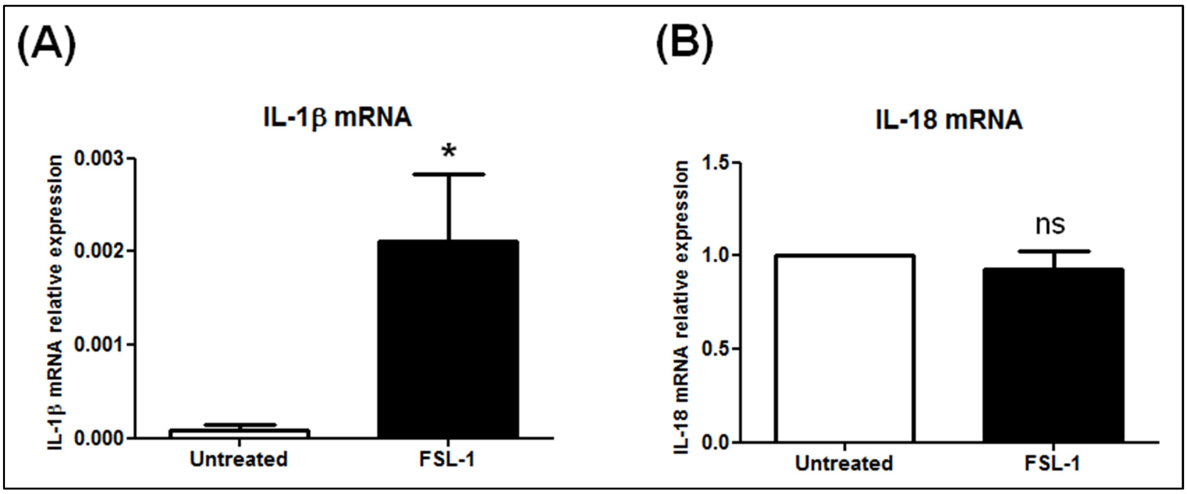

Supplementary figure 1: IL-1β but not IL-18 transcripts are increased in response to FSL-1 in AECs.

Transcripts expression of IL-1β (A) and IL-18 (B) was measured in AECs treated or not with 250ng/mL of FSL-1 for 4 hours.

Statistics: non-parametric T-test, Mann-Whitney test. ns: not significant; *: $p<0,05$.

AECs: amnion epithelial cells; IL: interleukin; FSL-1: fibroblast-stimulating lipopeptide-1.

SUPPLEMENTARY TABLES

Human gene	Forward sequence (5'→3')	Reverse sequence (5'→3')	Annealing temperature (°C) for RT-PCR	Annealing temperature (°C) for quantitative RT-PCR
ASC	AACGTGCTGCGCGACATGG	ACTGCCTGGTACTGCTCATC	61	61
Caspase-1	AGCTGAGGTTGACATCACAGG	TGTCAGAGGTCTTGTGCTCTG	64	62
Gasdermin D	AGCCTGCAGAGCTCCACTG	ATCGTAGAAGTGGAAGCTCC	61	58
IL-1β	AATCTCCGACCACCACTACAG	TCCCATGTGTCGAAGAAGATAG	64	62
IL-18	ATCAGATTACTTTGGCAAGCTTG	AGGCTGGCTATCTTTATACATAC	59	60
NLRP7	TGCTGTACAAGACCATGACACG	ACTCAAGCCCTCACACAGAAAC	62	61
RPLP0	AGGCTTTAGGTATCACCACT	GCAGAGTTTCCTCTGTGATA	ø	58, 61, 62
RPS17	TGCGAGGAGATCGCCATTATC	AAGGCTGAGACCTCAGGAAC	ø	58, 61, 62

Supplementary table 1: Forward and reverse primer sequences used for RT-PCR and RT-qPCR amplification of the human genes.

These primer sequences were used in order to amplify human cDNA of indicated genes obtained after reverse transcription of total mRNAs, extracted from human fetal membranes tissues or primary amnion cells.

ASC: apoptosis-associated speck-like protein containing a CARD domain; IL: interleukin; NLRP: nucleotide-binding oligomerization domain-like receptor, pyrin domain containing; RPLP0: ribosomal protein lateral stalk subunit P0; RPS17: ribosomal protein S17.

Antibody	Species (clonality)	Compagny (catalog number)	Dilution for western blotting	Dilution for immunofluorescence
Anti-ASC	Mouse (monoclonal)	Santa Cruz Biotechnology (sc-271054)	1:200	1:25
Anti-caspase-1	Rabbit (polyclonal)	Abcam (ab1872)	1:200	ø
Anti-NLRP7	Rabbit (polyclonal)	Thermofisher Scientific (PA5-21023)	1:300	1:500
Anti-gasdermin D	Rabbit (polyclonal)	Novus Biologicals (NBP2-33422)	1:400	ø

Supplementary table 2: Primary antibodies, their sources and uses.

ASC: apoptosis-associated speck-like protein containing a CARD domain; NLRP: nucleotide-binding oligomerization domain-like receptor, pyrin domain containing.

Species	Forward sequence (5'→3')	Reverse sequence (5'→3')	Annealing temperature (°C) for PCR
Mycoplasma fermentans	GGACTATTGTCTAAACAATTTCCC	GGTTATTCGATTTCTAAATCGCCT	62
Mycoplasma salivarium	ATGGATTGTAAAGTGCTGTTGCTAG	GCGTCAACAGTTCTCTGCCG	65

<u>Supplementary table 3:</u> Forward and reverse primer sequences used for PCR amplification of the mycoplasmas' gDNA.

These primer sequences were used in order to amplify the *Mycoplasma fermentans* and *Mycoplasma salivarium* gDNA from human amnion and choriodecidua samples.
